# Upstream sequence-dependent suppression and AtxA-dependent activation of protective antigens in *Bacillus anthracis*

**DOI:** 10.7717/peerj.6718

**Published:** 2019-04-12

**Authors:** Kochi Toyomane, Yoshikazu Furuta, Daisuke Fujikura, Hideaki Higashi

**Affiliations:** 1 Division of Infection and Immunity, Research Center for Zoonosis Control, Hokkaido University, Sapporo, Japan

**Keywords:** *Bacillus anthracis*, AtxA, Protective antigen, Gene silencing, Transcription factor

## Abstract

The anthrax toxin is a virulence factor produced by the bacterium *Bacillus anthracis*. Transcription of anthrax toxin genes is controlled by the transcription factor AtxA. Thus, AtxA is thought to be a key factor for the pathogenicity of *B. anthracis*. Despite its important role in *B. anthracis* infection, the molecular mechanism by which AtxA controls expression of anthrax toxin remains unclear. This study aimed to characterize the molecular mechanism of AtxA-mediated regulation of protective antigen (PA), a component of anthrax toxin encoded by the *pagA* gene. First, the interaction between the upstream region of *pagA* and AtxA was evaluated in vivo by constructing a transcriptional fusion of the upstream region with an auxotrophic marker. The results showed that (i) the upstream region of *pagA* suppressed transcription of the downstream gene and (ii) AtxA recovered suppressed transcription. Second, in vitro analysis using a gel mobility shift assay was performed to evaluate binding specificity of the AtxA–DNA interaction. The result showed sequence-independent binding of AtxA to DNA. Taken together, our findings suggest that the expression of PA was suppressed by the upstream region of *pagA* and that an interaction of AtxA and the upstream region releases the suppression.

## Introduction

*Bacillus anthracis*, a Gram-positive spore-forming bacterium, is the causative agent of the zoonotic infectious disease anthrax. The virulence of *B. anthracis* is determined by two large plasmids, namely pXO1 and pXO2, that encode major virulence factors. In particular, pXO1 encodes anthrax toxins while pXO2 encodes poly-γ-D-glutamate capsule synthetases (Mock & Fouet, 2001). Anthrax toxin is an A-B toxin composed of a protective antigen (PA), lethal factor (LF), and edema factor (EF). Lethal factor in combination with PA causes cell death, whereas EF in combination with PA induces edema.

The expression of these major virulence factors is regulated at the transcription level and is dependent on AtxA, a *B. anthracis*-specific protein encoded on pXO1 that positively regulates transcription of virulence genes (Fouet, 2010; Koehler, Dai & Kaufman-Yarbray, 1994; Uchida et al., 1993, 1997). Examples of genes controlled by AtxA are the toxin genes *pagA*, *lef*, and *cya*, which encode PA, LF, and EF, respectively (Koehler, Dai & Kaufman-Yarbray, 1994; Uchida et al., 1993). AtxA also controls the expression of pXO2-encoded capsule synthetases and chromosomal genes such as S-layer proteins (Guignot, Mock & Fouet, 1997; McKenzie et al., 2014; Mignot, Mock & Fouet, 2003; Uchida et al., 1997). Given that AtxA controls the expression of virulence factors, AtxA is related to the pathogenicity of *B. anthracis* (Dai et al., 1995). Furthermore, Dale et al. (2018) reported that AtxA level in *B. anthracis* is associated with sporulation and toxin production. *B. anthracis* generally expresses low amounts of AtxA in the sporulation phase, whereas cells in the vegetative condition produce AtxA at relatively higher levels. A *B. anthracis* mutant that constitutively overexpresses AtxA showed a sporulation defect even when in the preferable condition for sporulation. Thus, AtxA levels in cells may inversely influence the formation of spores and the production of virulence factors (Dale et al., 2018).

AtxA is thought to belong to a protein family related to the phosphotransferase system (PTS), which is a well-characterized regulatory system that controls the metabolism of carbohydrates in both gram-positive and gram-negative bacteria by transferring phosphoryl residues from one component to another (Barabote & Saier, 2005; Postma, Lengeler & Jacobson, 1993; Tsvetanova et al., 2007). Among the PTS-related proteins, a family of regulator proteins containing PTS regulation domains (PRDs) plays an important role in the control of metabolism (Görke & Stülke, 2008). One member of this transcription factor family is LicT, an RNA anti-terminator that regulates the expression of genes associated with β-glucoside metabolism, and LicT-mediated regulation is known to be dependent on the PTS (Graille et al., 2005; Van Tilbeurgh, Le Coq & Declerck, 2001). Regulatory proteins containing PRDs bind to specific DNA or RNA sequences to control the expression of specific genes. Recently, an emerging class of transcription factors controlling the transcription of virulence genes has been identified. This class of transcription factors, which are termed PRD-containing virulence regulators (PCVRs), includes AtxA in *B. anthracis*, Mga in *Streptococcus pyogenes,* and Mga*Spn* in *Streptococcus pneumoniae* (Hammerstrom et al., 2015; McIver et al., 1995; Solano-Collado, Espinosa & Bravo, 2012). These transcription factors share helix-turn-helix (HTH) DNA-binding domains as well as PRD domains and can regulate the expression of virulence genes. The detailed molecular structure of AtxA has been revealed by recent studies. As predicted, AtxA comprises five domains: two HTH domains, two PRD domains, and one enzyme II-like domain (Hammerstrom et al., 2015). It has also been shown that while PRD1 and PRD2, two PRD domains of AtxA, could be phosphorylated on AtxA-mediated virulence regulation (Tsvetanova et al., 2007). By using some AtxA mutants mimicking the phosphorylation or dephosphorylation status of histidine residues, Tsvetanova et al. (2007) and Hammerstrom et al. (2015) showed that phosphorylation of the histidine residue on PRD1 is necessary for AtxA-mediated transcriptional activation of toxin genes, while phosphorylation of that on PRD2 inhibits the transcription of toxin genes. The functional role of the phosphorylation on PRD1 remains unclear, though it is known that phosphorylation on PRD2 inhibits the function of AtxA by impairing dimerization of AtxA, which is necessary for activation of AtxA (Hammerstrom et al., 2011, 2015). Although there have been many studies on AtxA, the molecular mechanisms by which PCVRs control the expression of virulence genes are poorly understood due to the limited knowledge of interactions between PCVRs and DNA, though some studies have suggested sequence-independent binding of PCVRs (McIver et al., 1995; Solano-Collado et al., 2016). It has also been suggested that AtxA may bind to DNA with structural specificity rather than sequence specificity, although this hypothesis has not yet been supported by actual data (Fouet, 2010; Hadjifrangiskou & Koehler, 2008).

Despite its important role in *B. anthracis* infection, the molecular mechanism underlying the AtxA-dependent virulence regulation system remains unclear. To address this research gap, we investigated AtxA-dependent transcriptional upregulation of PA both in vivo and in vitro. The data presented here indicate that the upstream region of *pagA* suppresses transcription of downstream genes and that AtxA relaxes suppression by interacting with the upstream region in a sequence-independent manner.

## Materials and methods

### Bacterial strain and plasmids construction

Genomic DNA was extracted from overnight culture of *B. anthracis* str. CZC5 (Ohnishi et al., 2014) using a PowerFecal DNA isolation kit (MO BIO, Carlsbad, CA, USA) according to the manufacturer’s protocol. The *atxA* gene was amplified by polymerase chain reaction (PCR) using two primers with a restriction enzyme site, namely KpnI-AtxA-F and NheI-AtxA-R, and was then cloned into the pGEM-T-easy plasmid (Promega, Madison, WI, USA) according to manufacturer’s protocol (Table S1). After digestion by KpnI-HF (NEB, Ipswich, MA, USA) and NheI-HF (NEB, Ipswich, MA, USA), the full length of the *atxA* gene was inserted into a bait plasmid, pB1H2-ω2 (Addgene plasmid # 18038) (Noyes et al., 2008) and digested by KpnI-HF and XbaI (NEB, Ipswich, MA, USA); this construct expresses AtxA with the omega subunit of bacterial RNA polymerase. For the control transcription factor, pB1H2-ωL-Prd (Addgene plasmid # 18040), a bait plasmid expressing Paired, a well-characterized transcription factor, was used (Jun & Desplan, 1996). To construct a bait vector lacking the omega factor, the pB1H2-ωL vector was digested by EcoRI-HF (NEB, Ipswich, MA, USA) and XbaI, blunted using a DNA blunting kit (Takara, Kusatsu, Japan), and then ligated. For expression of AtxA *in trans*, where AtxA is expressed without the omega factor of RNA polymerase, the pSTV28 vector (Takara, Kusatsu, Japan) was used. The fragment of the *atxA* gene with the restriction enzyme sites was amplified by PCR using two primers from the pB1H2-ω2-AtxA vector, namely ERI-pB1H2-TF-F and BHI-AtxA-R. After digestion by EcoRI-HF and BamHI-HF (NEB, Ipswich, MA, USA), the amplicon was inserted into the expression vector pSTV28 and digested by the same enzymes.

To analyze the AtxA–DNA interaction, plasmids with the upstream sequence of *pagA*, here defined as *Ppag,* were constructed as prey. The primers used to amplify the upstream regions are shown in Table S1. After digestion by NotI-HF (NEB, Ipswich, MA, USA) and EcoRI-HF, the sequence was inserted into the prey vector pH3U3 (Addgene plasmid # 12609) (Meng, Brodsky & Wolfe, 2005). A total of 1,004 bp DNA fragment of lambda phage genome DNA was used as a control sequence. The DNA fragments with lambda phage DNA (sequence homology with the region from 17,817 to 18,798 bp in J02459.1, corresponding to partial sequence of host specificity protein J) were purified from one Kb Plus DNA Ladder (Invitrogen, Carlsbad, CA, USA) and introduced into the pH3U3 plasmid digested by EcoRI-HF after treatment with a DNA blunting kit. The full DNA sequence used as the control is available in Data S1. There was no significant similarity between *Ppag* and control DNA on BLAST alignment (Zhang et al., 2000). For construction of prey plasmids with partial *Ppag* sequences, pH3U3 plasmids with intact *Ppag* were amplified by the primers listed in Table S1, followed by digestion with EcoRI-HF (NEB, Ipswich, MA, USA), self-ligation with Ligation High ver2 (TOYOBO,Osaka, Japan), and transformation into *Escherichia coli* 10 β (NEB, Ipswich, MA, USA) with the heat shock method described elsewhere.

### Detection of protein–DNA interaction by transcriptional fusion with an auxotrophic marker

The *E. coli* strain US0 *hisB pyrF rpoZ* (Addgene # 18049) was transformed with bait pB1H2-ω2-AtxA, pB1H2-ωL-Prd, pB1H2(-), pSTV28-AtxA, or pSTV28 by the calcium chloride method (Meng, Brodsky & Wolfe, 2005; Meng & Wolfe, 2006; Noyes et al., 2008). The *E. coli* strain harboring bait plasmids was then treated with 10% glycerol for electroporation. Prey plasmids were subsequently introduced by electroporation (1,750 V, 25 μF, 200 Ω, one mm gap). The transformants were incubated for 60 min at 37 °C in one ml of Super Optimal broth with Catabolite repression (SOC) medium. Incubated cells were centrifuged and then resuspended in NM medium (Meng, Brodsky & Wolfe, 2005) supplemented with 200 μM uracil and 0.1% histidine. The cells were washed with NM medium four times and suspended in one ml of NM medium. From this suspension, five μl drops were spotted on NM plates without histidine after 10-fold serial dilution and incubated for 36 h at 37 °C. Transformation efficiency was determined using 2xYT plates, which include histidine, after overnight incubation at 37 °C. Given that the nutrient composition differs between histidine-deficient and histidine-rich conditions, colony size is larger in a histidine-rich condition using 2xYT plates even though the incubation period is shorter. For experiments using the pB1H2 vector, carbenicillin, and kanamycin were used as selection antibiotics for both 2xYT plates and NM plates. For experiments using the pSTV28 vector, chloramphenicol, and kanamycin were used as selection antibiotics for both 2xYT plates and NM plates. All antibiotics except for chloramphenicol were dissolved in water; chloramphenicol was dissolved in ethanol.

### Purification of HIS-FLAG-AtxA-HIS recombinant protein

To examine the interaction between AtxA and DNA sequences in vitro, the *atxA* gene was cloned into the expression vector pET 28-b (Novagen, Madison, WI, USA) with N- and C-terminal 6xHIS-tags and an internal FLAG-tag. The BL21 (DE3) expression strain of *E. coli* was then transformed with an AtxA expression vector. Cells harboring the AtxA expression vector were cultured at 37 °C in LB medium containing 50 μg/ml kanamycin. After overnight growth, cells were diluted to an OD600 of 0.05 in 200 ml of LB medium containing kanamycin and cultured to an OD600 of 0.5. Next, the cells were induced by adding one mM IPTG and cultured at 25 °C for 16 h. After incubation, the cells were collected by centrifugation at 10,000×*g* for 10 min and stored at −80 °C. Thawed cells were suspended in eight ml binding buffer (50 mM NaH_2_PO_4_, 300 mM NaCl, 0.5% Triton X-100, 10 mM imidazole, one mM PMSF, pH 8.0) and disrupted by sonication. After centrifugation for 15 min at 15,000×*g*, recombinant AtxA protein was purified by Ni-NTA (Qiagen, Hilden, Germany). The extract was then desalted by HiPrep 26/10 Desalting (GE, Boston, MA, USA) and purified by RESOURCE S, one ml (GE, Boston, MA, USA) using 25 mM MES, pH 6.5. The final concentration of recombinant AtxA was determined by a Bio-Rad Protein Assay (Bio-Rad, Hercules, CA, USA).

### In vitro gel mobility shift assay

Purified recombinant AtxA protein was serially diluted twofold from 200 μg/ml to 12.5 μg/ml. A total of 200 μg/ml of BSA was used as a control protein. Next, 6.6 μl protein solution was mixed to a final concentration of 20 ng/μl of *Ppag* or lambda phage genome DNA in 10 μl. For each DNA fragment, both the linear form prepared by PCR using HU100 and OK181v2 and the circular form cloned into pH3U3 were used. After 30 min at 37 °C, the mixture was electrophoresed using 1% agarose gel and 1xTAE buffer (Hellman & Fried, 2007). Ethidium bromide staining was used to detect DNA.

### In silico analysis

The guanine and cytosine (GC) contents in the DNA fragments were analyzed using GENETYX-MAC software (Network version 19.0.1; GENETYX Co., Tokyo, Japan). The average span for the analysis was set at 20 bp and the step was set at 1. DNA fragments were compared by dot plot analysis using dotmatcher (Rice, Longden & Bleasby, 2000). The window size was set at 10 and the threshold was set at 25.

## Results

### Transcription of *pagA* is suppressed by the upstream region and released by interaction with AtxA

**Figure 1:**
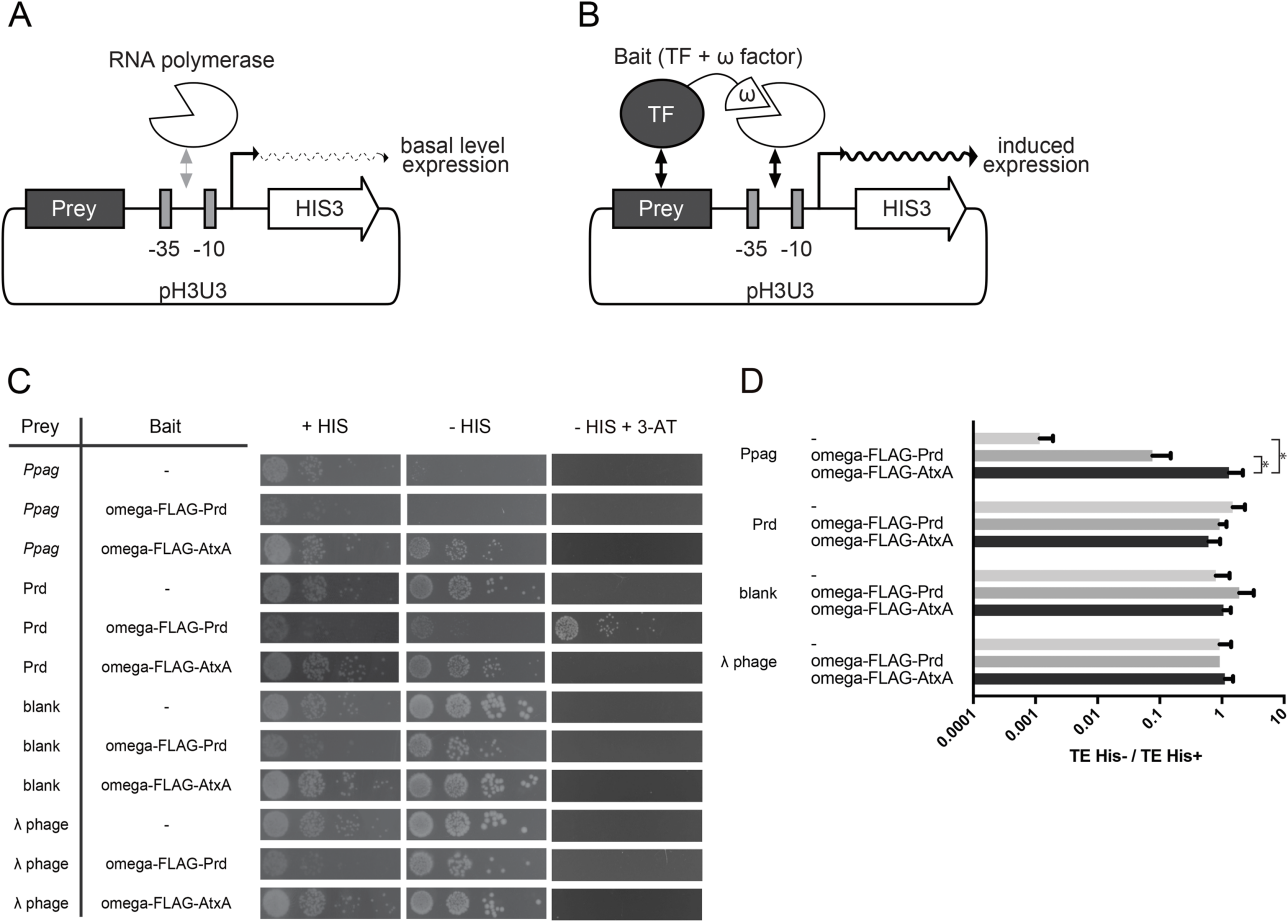
Detection of suppression by Ppag with the B1H system. (A, B) Scheme of B1H system for detection of binding of transcription factors to a specific DNA sequence. (A) When bait is absent, the HIS3 gene on the reporter plasmid is expressed at the basal level, which is sufficient for survival under conditions without histidine. (B) When specific bait is available in the same cell, the bait binds to the prey sequence and the omega factor fused to the bait protein recruits RNA polymerase molecules, resulting in enhanced HIS3 gene expression. (C) Colonies formed by the transformation of the reporter plasmid into the selection strain with each bait. For the -HIS3 and +3-AT medium, 5mM of 3-AT was supplemented. From left to right, each spot represents a 10-fold serial dilution of cells. (D) The ratio of transformation efficiency (TE) calculated from the colony numbers under conditions with or without histidine. *indicates *p* < 0.05 by the Mann–Whitney test.

To test the interaction between the upstream sequence of *pagA* (*Ppag*) and AtxA, we constructed a transcriptional fusion system with an auxotrophic marker, which was originally used in the Bacterial One-Hybrid (B1H) system (Fig. 1A) (Meng, Brodsky & Wolfe, 2005; Meng & Wolfe, 2006; Noyes et al., 2008). Although the B1H system was originally developed as a method for selection of sequences to which a eukaryotic transcription factor binds, it can also be used for selection of prokaryotic transcription factors that bind to specific sequences (Guo et al., 2009). The system consists of two vectors: (i) an expression plasmid harboring a gene encoding a bait protein and (ii) a reporter plasmid harboring a DNA region that works as prey. The bait protein is a transcription factor fused with a FLAG-tag and the omega subunit of a bacterial RNA polymerase. The prey is a DNA sequence that is a candidate of the binding site of the bait protein. The gene connected downstream of the prey sequence is the auxotrophic marker HIS3 gene, whose product is required for survival in the condition lacking histidine. When the bait protein stably binds to the prey sequence, the fused omega subunit recruits the whole bacterial RNA polymerase, resulting in the induction of HIS3 expression. Although the basal level of HIS3 expression without the bait protein is sufficient for growth in the condition without histidine, the higher level of HIS3 expression induced by the bait protein is required for survival when the culture that lacks histidine is treated with 3-amino-triazole (3-AT), the HIS3 competitive inhibitor (Fig. 1A). By using 3-AT, the system can select the combination of bait and prey that interact above a certain threshold, resulting in higher expression of HIS3 and survival under the chosen concentration of 3-AT. The system can also be utilized for measuring the interaction between a specific combination of bait protein and prey sequence by measuring the survival rate of strains introduced by plasmids encoding the bait and prey under conditions with or without histidine and 3-AT.

We adopted this system to investigate the interaction between the upstream sequence of the *pagA* gene and AtxA. AtxA fused with a FLAG-tag and the omega subunit of bacterial RNA polymerase was constructed using the expression plasmid as a bait, while *Ppag* was fused upstream of HIS3 gene on the reporter plasmid as prey. The strain harboring the expression plasmid was transformed with the reporter plasmid with *Ppag* and the transformation efficiency ratio, which is the transformation efficiency without histidine divided by that with histidine, was calculated to test the expression of HIS3. The expected transformation efficiency ratio was observed when the strain harboring the expression plasmid encoding AtxA was transformed with the reporter plasmid with *Ppag*. However, a significantly lower transformation ratio was noted when the strain without AtxA bait was transformed with the reporter plasmid with *Ppag* (Figs. 1B and 1C). These observations indicate that, without AtxA, the expression level of HIS3 transcriptionally fused with *Ppag* was lower than the basal level and insufficient for survival in the condition without histidine. We hypothesized that *Ppag* suppressed the expression of the downstream gene when AtxA was absent, and that suppression was released by the interaction between *Ppag* and AtxA. Suppression without bait was not observed as expected when we used Prd as the bait and the Prd binding motif as the prey, which is a known combination of bait and prey with a strong interaction (Meng & Wolfe, 2006) (Figs. 1B and 1C).

To validate our system, we also tested the growth of the strains under the condition with 3-AT but in the absence of histidine. Although the combination of Prd and the Prd binding motif showed growth under the condition with 3-AT, the strain expressing AtxA and harboring *Ppag* did not show any growth with 3-AT (Fig. 1B). Therefore, the results suggested that AtxA could recover the growth of the strain harboring *Ppag* but that AtxA could not induce HIS3 expression to a level that would allow bacteria to grow on the plate with 3-AT. The reporter plasmid with the Prd binding motif showed the same transformation efficiency when transformed to strains with and without Prd as bait. In addition, the introduction of Prd as bait did not release suppression by *Ppag*, suggesting that AtxA-specific interactions with *Ppag* are required to release suppression (Figs. 1B and 1C).

To test if suppression was caused by any upstream sequence, two reporter plasmids were constructed as additional controls: one was a reporter plasmid without any additional sequence insertion upstream of the marker, and the other was a plasmid inserted with a partial sequence of the lambda phage. Those plasmids were transformed into strains with either Prd or AtxA as the bait and the strain without bait, but they showed no differences in transformation efficiency (Figs. 1B and 1C). This supports our hypothesis that suppression was specifically caused by *Ppag*. Suppression by *Ppag* might be partly explained by the AT-rich profile of *Ppag* relative to that of the lambda phage (GC contents of 24.5% for *Ppag* vs. 54% for lambda phage DNA, see also Fig. S1), as early studies suggested that AT-rich regions negatively affect transcription in *E. coli* (Navarre et al., 2006). This is consistent with an earlier report suggesting a functional relationship between AtxA and AT-rich regions of DNA (Hadjifrangiskou & Koehler, 2008).

**Figure 2:**
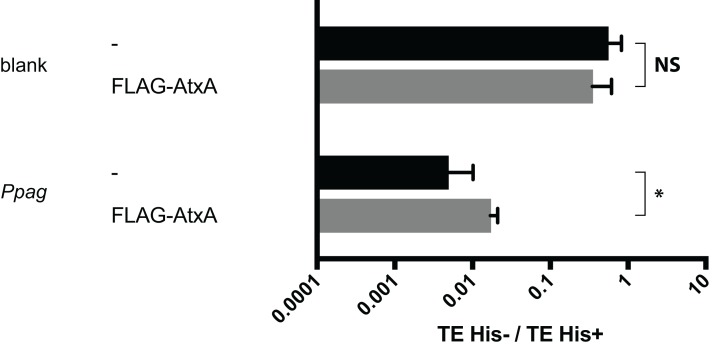
Recovery from suppression by *Ppag* without omega factor. Interaction of the FLAG-AtxA with the upstream region of *pagA* gene was compared with that of an empty vector. A pSTV28 vector expressing no bait protein was used as a control (-). *Indicates *p* < 0.05 and NS indicates a non-significant difference, both by the Mann–Whitney test.

Binding of AtxA to upstream regions was also assessed using a vector expressing AtxA without the omega factor to determine whether AtxA-dependent growth recovery required the omega factor fused to AtxA. A strain with an expression plasmid expressing FLAG-AtxA, in which AtxA is fused with a FLAG-tag but without the omega factor, was constructed. The transformation assay for this strain using reporter plasmids with and without *Ppag* showed that FLAG-AtxA significantly recovered growth inhibition (Fig. 2; Fig. S2). This result indicated that, together with the recovery effect of the omega-AtxA fusion protein and omega-Prd fusion protein (Figs. 1B and 1C), AtxA and the omega factor likely recover transcription in an additive manner.

**Figure 3:**
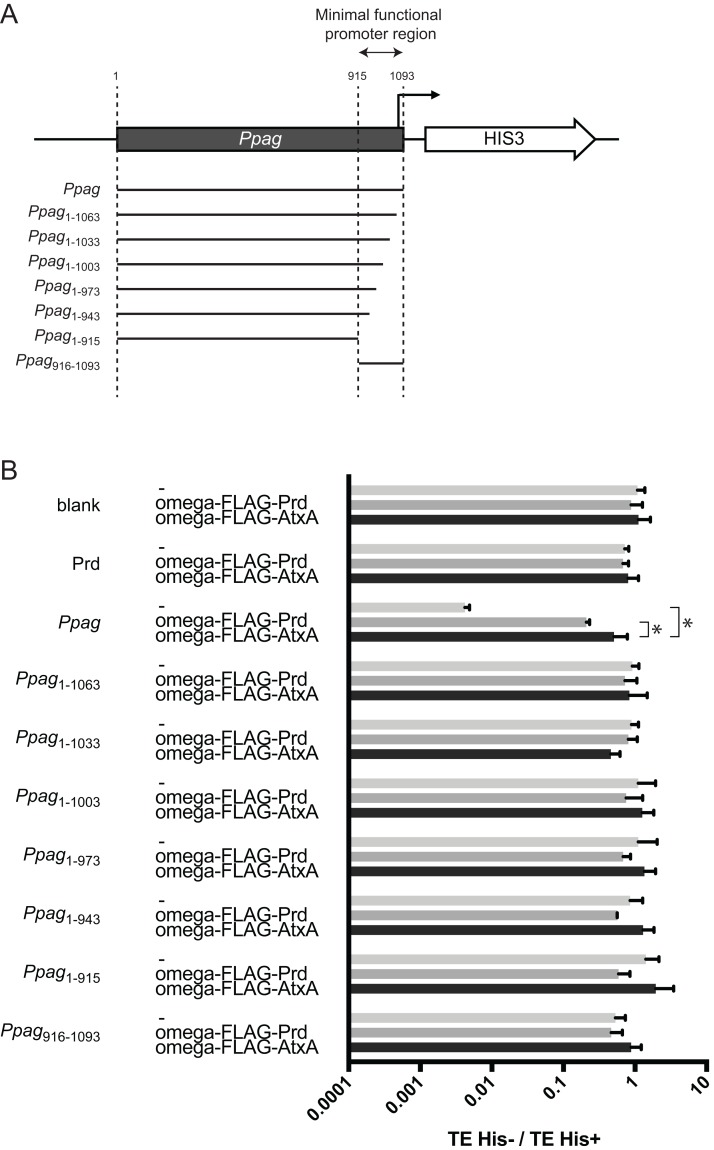
Determination of the responsible region for suppression by *Ppag*. Interaction of omega-FLAG-AtxA with full-length *Ppag* was compared to that with deleted variants of *Ppag*. (A) Schematic figure showing the deleted variants of *Ppag.* (B) The ratio of transformation efficiency between conditions with or without histidine. A bait vector expressing omega-FLAG-Prd was used as a control transcription factor. *Indicates *p* < 0.05 by the Mann–Whitney test.

Hadjifrangiskou & Koehler (2008) predicted that the minimal functional promoter region for AtxA-dependent transcriptional regulation of *pagA* transcription is a 178 bp sequence of the *pagA* promoter region (Fig. 3A). We first tested if this predicted minimal functional promoter, here defined as *Ppag*_916-1093_, contributed to suppression by *Ppag*. Surprisingly, *Ppag*_916-1093_ as well as *Ppag*_1-915_, which lacks the minimal functional promoter region, did not affect transformation efficiency (Fig. 3; Fig. S3). This suggests that coexistence of the minimal functional promoter region and the other region of *Ppag* are required for the suppression observed with whole length *Ppag*. To identify the region in the minimal functional promoter responsible for the suppression by *Ppag*, we then constructed a reporter plasmid with the *Ppag* sequence with stepwise 30 bp deletions from the 3′ end in the minimal functional promoter region (Fig. 3A). The results indicated that deletion of 30 bp from the 3′ end of *Ppag* was sufficient to eliminate suppression (Fig. 3B; Fig. S3), suggesting that the 30 bp end is one of the core regions contributing to suppression by the whole *Ppag* sequence.

### In vitro analysis of AtxA–DNA interaction

**Figure 4:**
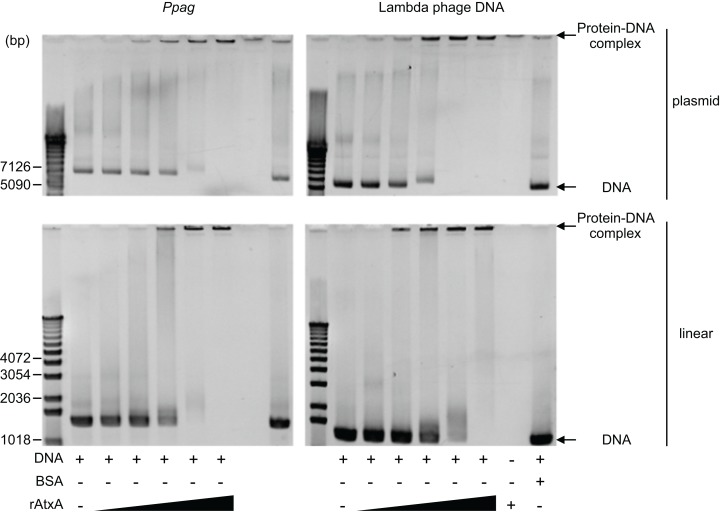
AtxA-DNA binding in a low sequence-specific manner sequence. *Ppag* or lambda phage DNA fragments were incubated with increasing concentrations (12.5–200 μg/ml) of recombinant AtxA protein or 200 μg/ml of BSA in a plasmid or linear form. Free DNA and protein–DNA complex were separated by 1% agarose gel. Bands corresponding to free DNA and protein–DNA complexes are indicated.

To determine whether recovery of AtxA depends on the specificity of the AtxA–DNA interaction, we tested the binding specificity of AtxA to *Ppag* in vitro. Recombinant full-length AtxA was expressed in *E. coli* as a HIS-tagged protein and purified using Ni-NTA agarose and a Resource S column (Fig. S4). Recombinant AtxA was incubated with DNA fragments with the *Ppag* sequence or the partial lambda phage sequence as a control, and then the interaction was tested using an electrophoretic mobility shift assay (EMSA). To detect the interaction of AtxA with long DNA, we used agarose gel for EMSA (Hellman & Fried, 2007). For both *Ppag* and lambda phage DNA, we observed DNA bands stuck in the wells with recombinant AtxA at concentrations of 50–100 μg/ml, which is suggestive of AtxA-DNA binding (Fig. 4). DNA substrates in both the plasmid form and linear form were tested and showed similar results, suggesting that the form of the DNA substrate does not affect the interaction with AtxA. Considering the low sequence similarity between *Ppag* and lambda phage DNA (see Materials and Methods and Fig. S5), the similar binding profile between *Ppag* and lambda phage DNA suggests that the binding of recombinant AtxA to DNA fragments is not likely to be affected by the DNA sequence. Despite positive results obtained in in vitro binding assay, we observed no growth improvement in the in vivo B1H experiment when lambda phage DNA was used as prey and AtxA as a bait (Figs. 1B and 1C). In this study, we used recombinant AtxA with N- and C-terminal 6xHIS-tags and an internal FLAG-tag to analyze AtxA–DNA interaction. While both 6xHIS-tags and FLAG-tag are not likely to affect the function of AtxA, further study using a tag-free AtxA protein will be needed for further understanding the AtxA–DNA interaction.

## Discussion

In this study, we investigated the regulation of transcription of *pagA*, a component of the anthrax toxin gene. We found that *Ppag*, an upstream sequence of *pagA*, greatly suppresses the transcription of the downstream gene. We also found that AtxA relieves *Ppag*-mediated transcriptional repression and showed that AtxA likely binds to DNA without or with low sequence specificity regardless of functional specificity. This study is the first to suggest a competitive mechanism between the suppression of transcription mediated by the *pagA* upstream region of AT-rich sequence and a recovery effect mediated by AtxA. In bacterial genomes, the upstream region of an operon often contains functional genetic elements such as enhancers and silencers (Chen et al., 2001; Kulaeva et al., 2012). An enhancer is a genetic element that activates transcription *in trans* by recruiting activator protein, whereas a silencer is a genetic element responsible for gene silencing. Both bacterial enhancer and silencer elements have been shown to have AT-rich sequences. Insertion of a 72 bp AT-rich sequence (GC content of 22%) called AT4 was shown to repress transcription of the downstream gene as a bacterial gene silencer in *Salmonella typhimurium* (Chen et al., 2001). Recently, it was found that AT4 consists of two elements, namely AT7 and AT8, with opposing effects on the *pleuO* promoter (Chen et al., 2003): AT7 is responsible for derepression, while AT8 is responsible for repression. AT8-mediated gene silencing is derepressed by LeuO, the leucine biosynthesis regulator, which binds to AT7. Such AT-rich DNA elements with opposing effects are also found in *E. coli* and *Mycobacterium,* but with low sequence homology to those in *Salmonella*, suggesting that bacterial gene silencing systems are widespread (Chen et al., 2005; Gordon et al., 2010). In the present study, the AT-rich sequence *Ppag* also suppressed transcription of the downstream gene, suggesting the repressing effect of *Ppag* as a bacterial gene silencer.

Nucleoid-associated proteins (NAPs) have been shown to play important roles in transcriptional/post-transcriptional regulation systems by changing the conformation of local DNA structure (Dillon & Dorman, 2010). One characteristic of NAPs in various bacterial species is their ability to bind to DNA without well-conserved consensus sequences, although NAPs prefer some AT-rich or curved DNA areas (Gordon et al., 2010; Navarre et al., 2006; Owen-Hughes et al., 1992). Among *Bacillus* species, AbrB, an NAP originally found in *B. subtilis*, acts as a “transition regulator” (O’Reilly & Devine, 1997). It should be noted that AbrB negatively regulates the expression of anthrax toxin genes and *atxA* during the exponential growth phase (Saile & Koehler, 2002; Strauch et al., 2005). It has been shown that the repression element of the *leuO* gene is a binding site of a histone-like nucleoid-structuring protein (H-NS), the most extensively studied NAP, in both *S. typhimurium* and *E. coli* (Chen et al., 2005). Histone-like nucleoid-structuring protein binds to AT-rich DNA to silence transcription, forming a multimeric complex (Ceschini et al., 2000; Navarre et al., 2006). Since *Ppag* is highly AT-rich relative to pXO1 (GC content of 24.5% for *Ppag* and 32.5% for pXO1) (Okinaka et al., 1999), *Ppag* DNA could be the target of negative regulation factors that prefer AT-rich regions. As already mentioned, the LeuO protein activates the *pleuO* promoter by antagonizing the repressive effect of H-NS (Chen et al., 2003, 2005). As several factors can activate genes repressed by H-NS using various strategies (Dillon & Dorman, 2010; Stoebel, Free & Dorman, 2008), activation of transcription induced by AtxA could result from competition over recognition sites with NAPs, the repressive proteins that can bind to silencers.

Interestingly, Mga*Spn,* a PVCR member from *Streptococcus* *pneumoniae*, shows a binding profile similar to that of H-NS from *E. coli*, although these two proteins are structurally unrelated (Solano-Collado et al., 2016). Despite this similarity, the functions of these two proteins are opposite: transcription of genes targeted by Mga*Spn* is typically positively regulated, whereas that targeted by H-NS is typically negatively regulated (Navarre et al., 2006; Solano-Collado, Espinosa & Bravo, 2012). As a PCVR, AtxA shares two conserved DNA-binding domains with Mga*Spn* with 42.2% similarity and 21.3% identity (Solano-Collado, Espinosa & Bravo, 2012; Tsvetanova et al., 2007). Furthermore, AtxA functions as a positive regulator of virulence genes, which is similar to the function of Mga*Spn* (Solano-Collado, Espinosa & Bravo, 2012). Based on our results, we speculate that AtxA enhances the transcription of *pagA* by counteracting negative regulation factors similar to H-NS, such as AbrB. Despite recent advances in understanding of PVCR–DNA interactions, the binding profile of AtxA remained unclear. We thus tried to test if AtxA-DNA binding was sequence-dependent or sequence-independent. With increasing concentrations of recombinant AtxA, we observed more DNA bands stuck in the wells during electrophoresis. This finding is similar to that observed for EMSA in studies of LrpC, a DNA-binding protein of *B. subtilis* (Beloin et al., 2003; López-Torrejón, Martínez-Jiménez & Ayora, 2006), where LrpC was bound to both linearized dsDNA and circular DNA in a sequence-independent manner. Since stacking of DNA bands in the wells was also observed with an increasing concentration of AtxA, AtxA likely binds to both forms of DNA. Furthermore, AtxA bound to both *Ppag* and lambda phage DNA, which has no sequence similarity, suggesting that the AtxA–DNA interaction is likely sequence-nonspecific. Although transcriptional recovery by AtxA was specific to *Ppag*, AtxA likely binds DNA in a sequence-independent manner. This finding implies there are other factors that AtxA recognizes, such as DNA conformation.

The results of EMSA appeared contradictive as bait–prey interaction should enhance the transcription of HIS3 in the original B1H system. Further studies are needed to confirm the sequence-nonspecific AtxA–DNA interaction, as the recombinant AtxA with tag sequences potentially causes an artificial effect in in vitro binding experiments. On the other hand, these phenomena might be explained by the counteracting function of AtxA. Our results and results of earlier studies support the sequence-independent AtxA–DNA interaction. Therefore, it is thought that AtxA possibly binds to both *Ppag* and lambda phage DNA even in our in vivo system, considering the AtxA–DNA interaction observed in EMSA. However, the AtxA-lambda phage DNA showed no improvement of bacterial growth, suggesting that the AtxA-mediated improvement of bacterial growth observed with the strain expressing AtxA and harboring *Ppag* was not by the mechanism expected in the original B1H system in which specific bait–prey interactions with high affinity lead to more HIS3 expression. Rather, it is thought that AtxA likely acts as an antisilencer, such as LeuO (Chen et al., 2003; Stoebel, Free & Dorman, 2008).

The 178-bp sequence of the *pagA* promoter which is predicted as the minimal functional promoter of the *pagA* gene (Hadjifrangiskou & Koehler, 2008) did not show any effects on HIS3 expression in the transcriptional fusion system. This unexpected result might be explained by the in vivo system used in the present study. Though the predicted minimal functional promoter is able to initiate the transcription in *B. anthracis*, it is likely to be inactive in *E. coli* because of the differences in transcription systems in these species. In addition, the 178-bp fragment predicted as minimal functional promoter did not show a negative effect on bacterial growth. This result suggests that the phenomenon observed in our study is different from the typical transcriptional initiation.

Taken together, our results suggest that the functional specificity of AtxA is determined not by DNA sequence specificity but by factors like the architecture of bacterial DNA, including the conformation of DNA or other DNA-binding proteins. Although our study used *E. coli,* which is a heterologous host, the findings suggest that AtxA regulates the transcription of the *pagA* gene by relief of *Ppag*-mediated transcriptional repression. Further analysis of the molecular mechanisms of the AtxA-dependent virulence regulation system is required to better understand the function of PVCRs in pathogenesis.

## Conclusions

In conclusion, we found that *Ppag*, the upstream region of an anthrax toxin protein, negatively affected transcription of the downstream gene and that AtxA, a transcription factor of *B. anthracis*, recovered transcriptional inhibition by *Ppag*. This is the first report suggesting that a competitive mechanism underlies the AtxA-mediated transcriptional regulation system. Considering the sequence-independent binding of AtxA shown by EMSA, site-specific activation mediated by AtxA is likely determined by other factors that are unknown at this time.

## Supplemental Information

10.7717/peerj.6718/supp-1 Supplemental Information 1 GC content of DNA fragments used in this study. This figure was produced by GENETYX software with the average span set at 20. The shaded region on the *Ppag* panel indicates *Ppag*_916-1093_.

10.7717/peerj.6718/supp-2 Supplemental Information 2 Colonies observed for detection of recovery from suppression by *Ppag* without the omega factor.

10.7717/peerj.6718/supp-3 Supplemental Information 3 Colonies observed for determination of the responsible region for suppression by *Ppag*.

10.7717/peerj.6718/supp-4 Supplemental Information 4 Purification of recombinant AtxA protein. AtxA protein was expressed with 6xHIS tags and a FLAG-tag and then purified (see Method). The purified protein was analyzed by SDS-PAGE with CBB staining (A) or Western blotting (B) to detect the FLAG-tag. For recombinant AtxA, extract obtained just after sonication (Input) or after all purification procedures (rAtxA on CBB stain and Elute on Western blotting) was used.

10.7717/peerj.6718/supp-5 Supplemental Information 5 Dot plot analysis of DNA fragments used in this study. The DNA fragments used in this study were compared to each other using dotmatcher (http://emboss.bioinformatics.nl/cgi-bin/emboss/dotmatcher).

10.7717/peerj.6718/supp-6 Supplemental Information 6 Primers used in this study.

10.7717/peerj.6718/supp-7 Supplemental Information 7 DNA sequence of control DNA.

## Additional Information and Declarations

### Data Availability

The following information was supplied regarding data availability: The raw measurements for Figs. 2 and 3 are available in the Supplemental Files.
