# Supplementary figures and images for: Upstream sequence-dependent suppression and AtxA-dependent activation of protective antigens in Bacillus anthracis
Source: PeerJ. 2019 Apr 12;7:e6718. doi: 10.7717/peerj.6718 (PMC6463858; doi:10.7717/peerj.6718)

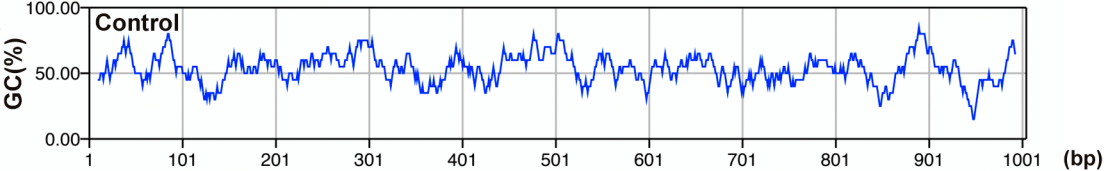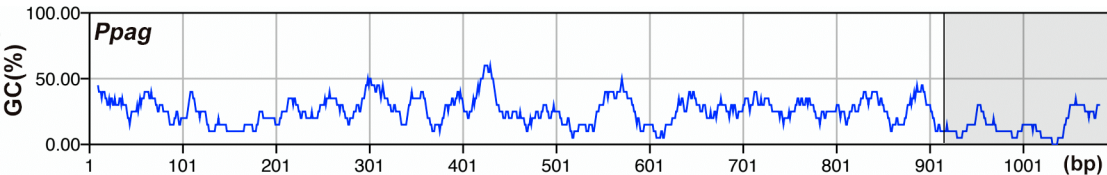

Supplement: Supplemental Information 1 — This figure was produced by GENETYX software with the average span set at 20. The shaded region on the Ppag panel indicates Ppag916-1093. [file peerj-07-6718-s001.pdf]

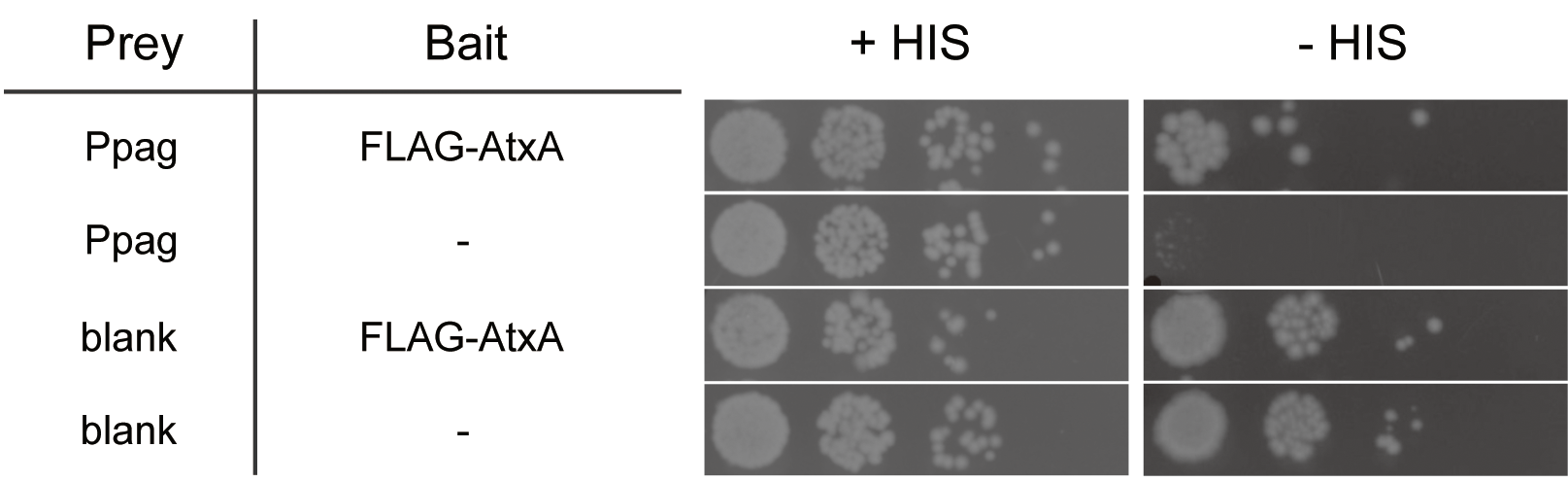

Supplement: Supplemental Information 2 [file peerj-07-6718-s002.png]

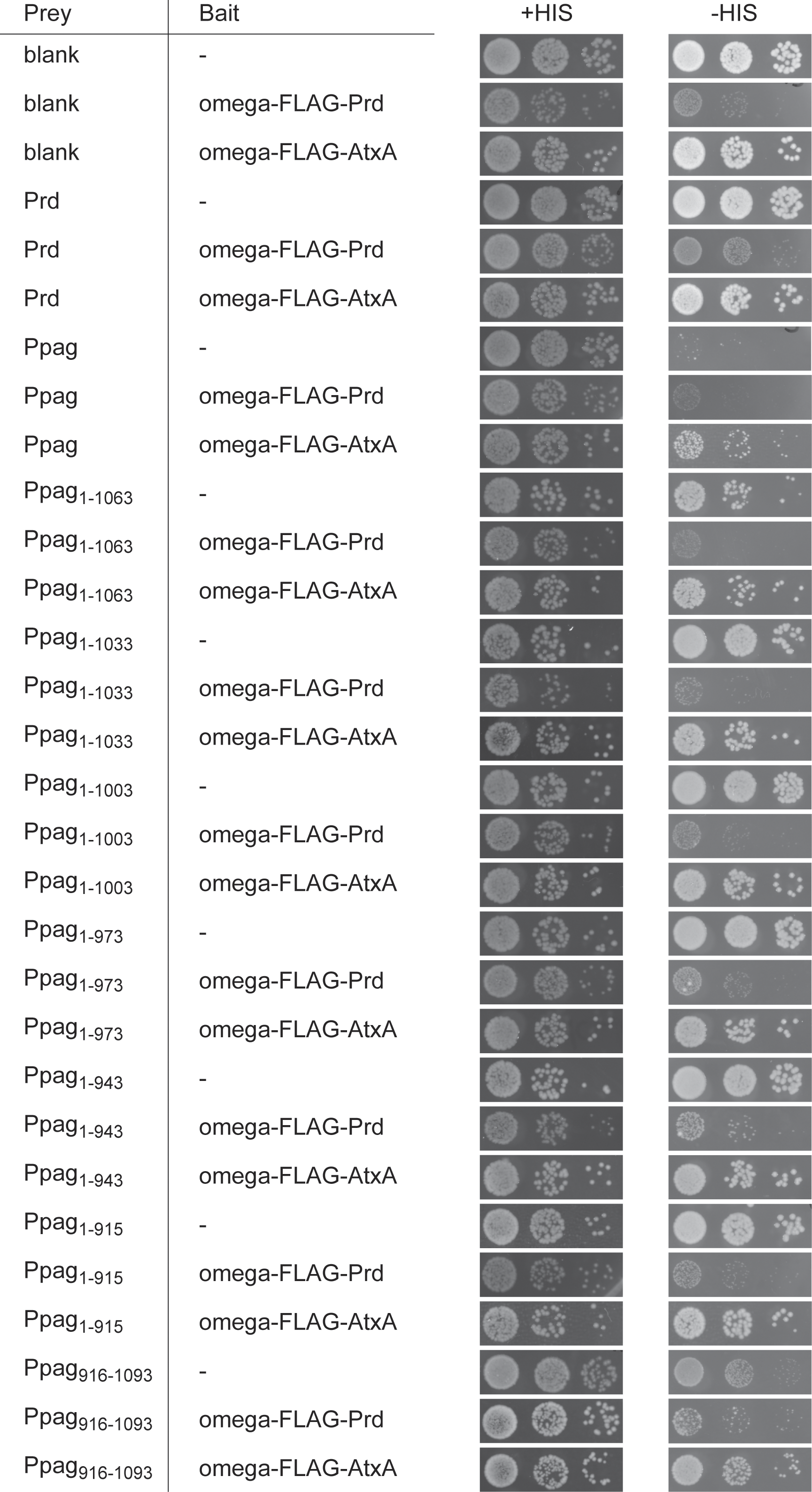

Supplement: Supplemental Information 3 [file peerj-07-6718-s003.png]

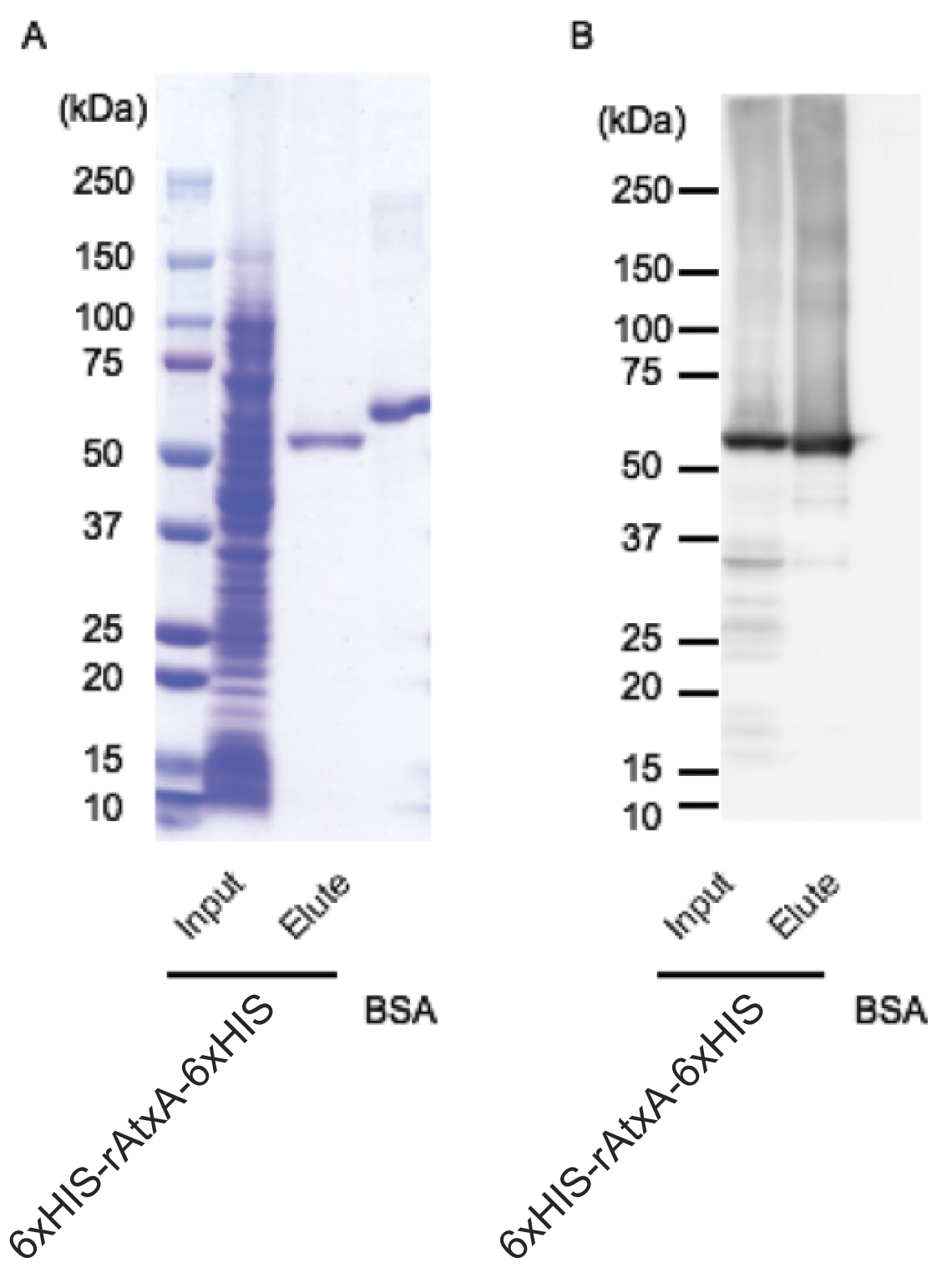

Supplement: Supplemental Information 4 — AtxA protein was expressed with 6xHIS tags and a FLAG-tag and then purified (see Method). The purified protein was analyzed by SDS-PAGE with CBB staining (A) or Western blotting (B) to detect the FLAG-tag. For recombinant AtxA, extract obtained just after sonication (Input) or after all purification procedures (rAtxA on CBB stain and Elute on Western blotting) was used. [file peerj-07-6718-s004.png]

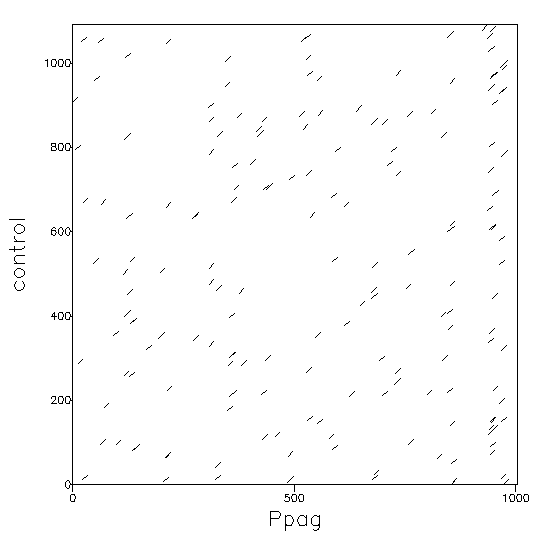

Supplement: Supplemental Information 5 — The DNA fragments used in this study were compared to each other using dotmatcher (http://emboss.bioinformatics.nl/cgi-bin/emboss/dotmatcher). [file peerj-07-6718-s005.png]
