# Supplementary material for: Upstream sequence-dependent suppression and AtxA-dependent activation of protective antigens in Bacillus anthracis
Source: PeerJ. 2019 Apr 12;7:e6718. doi: 10.7717/peerj.6718 (PMC6463858; doi:10.7717/peerj.6718)
Supplement: Supplemental Information 6 [file peerj-07-6718-s006.docx]

| Supplementary Table S1. Primers used in this study | | | |
| --- | --- | --- | --- |
| Name | Sequence (5' -> 3') | Use | Reference |
| KpnI-AtxA-F | GGGGTACCATGCTAACACCGATATCCATCG | Construction of vector expressing recombinant AtxA |  |
| NheI-AtxA-R | CCCGCTAGCTTATATTATCTTTTTGATTTCATG | Construction of vector expressing recombinant AtxA |  |
| NOTI-MH58s | GCGGCCGCAATTCTTTTTTATGTTATATATTTAT | Cloning of promoter regions | Hadjifrangiskou & Koehler 2008, modified |
| ERI-MH57as | GAATTCAATGGTATTAACACTTTTC | Cloning of promoter regions | Hadjifrangiskou & Koehler 2008, modified |
| NOTI-PpagL-F | GCGGCCGCGTAGATCCTGGTGATGATG | Cloning of promoter regions |  |
| PpagLd3R | TATATGTTTAATAGAAAAGGACACAGAAGC | Cloning of promoter regions |  |
| pB1H2wL-omega(-)-rev | GAATGCGGCCGCCATGGTGTCCTCTCTTTG | Deletion of omega factor from pB1H2wL vector |  |
| pB1H2wL-omega(-)-for | GTCGTGCGGCCGCGGACTACAAGGATGAC | Deletion of omega factor from pB1H2wL vector |  |
| ERI-FLAG-ATXA-F | GATCCGAATTCGGACTACAAGGATGACGAC | Construction of vector expressing recombinant AtxA |  |
| SACI-ATXA-R | ACGGAGCTCAATATTATCTTTTTGATTTCA | Construction of vector expressing recombinant AtxA |  |
| ERI-pB1H2-TF-F | TACGAATTCGGACTACAAGGATGACGACG | Construction of vector expressing recombinant AtxA |  |
| BHI-AtxA-R | TAGAGGATCCTTATATTATCTTTTTGATTTCA | Construction of vector expressing recombinant AtxA |  |
| OK181 | CCAGAGCATGTATCATATGGTCCAGAAACC | Sequencing primer | Meng & Wolfe 2006, modified |
| HU100 | CAAATATGTATCCGCTCATGAC | Sequencing primer | Meng & Wolfe 2006 |
