# Supplementary material for: Upstream sequence-dependent suppression and AtxA-dependent activation of protective antigens in Bacillus anthracis
Source: PeerJ. 2019 Apr 12;7:e6718. doi: 10.7717/peerj.6718 (PMC6463858; doi:10.7717/peerj.6718)
Supplement: Supplemental Information 7 [file peerj-07-6718-s007.docx]

Supplementary Data S1

>control DNA

GATCTGAATTCCGTGAACGTCAGGATCACGTTTCCCCGACCCGCTGGCATGTCAACAATACGGGAGAACACCTGTACCGCCTCGTTCGCCGCGCCATCATAAATCACCGCACCGTTCATCAGTACTTTCAGATAACACATCGAATACGTTGTCCTGCCGCTGACAGTACGCTTACTTCCGCGAAACGTCAGCGGAAGCACCACTATCTGGCGATCAAAAGGATGGTCATCGGTCACGGTGACAGTACGGGTACCTGACGGCCAGTCCACACTGCTTTCACGCTGGCGCGGAAAAGCCGCGCTCGCCGCCTTTACAATGTCCCCGACGATTTTTTCCGCCCTCAGCGTACCGTTTATCGTACAGTTTTCAGCTATCGTCACATTACTGAGCGTCCCGGAGTTCGCATTCACACTGCCACTGATATCCGCATTTTTAGCGGTCAGCTTTCCGTCCGGTGTCAGGGAAAAGGCCGGAGGATTGCCGCCGCTGGTAATGGTGGGGGCCGTCAGGCGCTTCAGGAACACGTCGTTCATGAATATCTGGTTGCCCTGCGCCACAAACATCGGCGTTTCATTCCCGTTTGCCGGGTCAATAAATGCGATACGATTGGCGGCAACCAGAAACTGGCTCAGTTTGCCTTCCTCCGTGTCCTCCATGCTGAGGCCAATACCCGCGACATAATGTTTGCCGTCTTTGGTCTGCTCAATTTTGACAGCCCACATGGCATTCCACTTATCACTGGCATCCTTCCACTCTTTCGAAAACTCCTCCAGTCTGCTGGCGTTATCCTCCGTCAGCTCGACTTTTTCCAGCAGCTCCTTGCCGAGATGGGATTCGGTTATCTTGCCTTTGAAAAAATCCAGGTAACCTTCCGCATCATCGCTCGCCCGACCGACGGCCTCCACGAATGCCGATTTGCCAACGGTGTTCACACTGCGGATATAAAAGTAATAATCATGGCCCGGTTTGATATTGATACTGGCGGCTATCCAGCCCGAGGGATC
